# Involvement of GTA protein NC2β in Neuroblastoma pathogenesis suggests that it physiologically participates in the regulation of cell proliferation

**DOI:** 10.1186/1476-4598-7-52

**Published:** 2008-06-06

**Authors:** Cinzia Di Pietro, Marco Ragusa, Davide Barbagallo, Laura R Duro, Maria R Guglielmino, Alessandra Majorana, Veronica Giunta, Antonella Rapisarda, Elisa Tricarichi, Marco Miceli, Rosario Angelica, Agata Grillo, Barbara Banelli, Isabella Defferari, Stefano Forte, Alessandro Laganà, Camillo Bosco, Rosalba Giugno, Alfredo Pulvirenti, Alfredo Ferro, Karl H Grzeschik, Andrea Di Cataldo, Gian P Tonini, Massimo Romani, Michele Purrello

**Affiliations:** 1 Dipartimento di Scienze Biomediche, Sezione di Biologia Generale, Biologia Cellulare, Genetica Molecolare *G Sichel*, Unità di Biologia Genomica e dei Sistemi Complessi, Genetica, Bioinformatica, Università di Catania, 95123 Catania, Italy; 2 Labogen, 95124 Catania, Italy; 3 Istituto Nazionale per la Ricerca sul Cancro (IST), Sezione di Genetica dei Tumori, 16132 Genova, Italy; 4 Istituto Nazionale per la Ricerca sul Cancro (IST), Sezione di Oncologia Traslazionale Pediatrica, 16132 Genova, Italy; 5 Dipartimento di Matematica ed Informatica, Università di Catania, 95123 Catania, Italy; 6 Medizinisches Zentrum für Humangenetik, Philipps Universität, 35037 Marburg, Germany; 7 Dipartimento di Pediatria, Università di Catania, 95123 Catania, Italy

## Abstract

**Background:**

The General Transcription Apparatus (GTA) comprises more than one hundred proteins, including RNA Polymerases, GTFs, TAFs, Mediator, and cofactors such as heterodimeric NC2. This complexity contrasts with the simple mechanical role that these proteins are believed to perform and suggests a still uncharacterized participation to important biological functions, such as the control of cell proliferation.

**Results:**

To verify our hypothesis, we analyzed the involvement in Neuroblastoma (NB) pathogenesis of GTA genes localized at 1p, one of NB critical regions: through RT-PCR of fifty eight NB biopsies, we demonstrated the statistically significant reduction of the mRNA for NC2β (localized at 1p22.1) in 74% of samples (p = 0.0039). Transcripts from TAF13 and TAF12 (mapping at 1p13.3 and 1p35.3, respectively) were also reduced, whereas we didn't detect any quantitative alteration of the mRNAs from GTF2B and NC2α (localized at 1p22-p21 and 11q13.3, respectively). We confirmed these data by comparing tumour and constitutional DNA: most NB samples with diminished levels of NC2β mRNA had also genomic deletions at the corresponding locus.

**Conclusion:**

Our data show that NC2β is specifically involved in NB pathogenesis and may be considered a new NB biomarker: accordingly, we suggest that NC2β, and possibly other GTA members, are physiologically involved in the control of cell proliferation. Finally, our studies unearth complex selective mechanisms within NB cells.

## Background

Transcription initiation, the most important and regulated event along the pathway connecting genotype to phenotype, is governed by the General Transcription Apparatus (GTA): GTA proteins constitute the PreInitiation Complex (PIC) and guide its assembly [1-3]. Class II PIC, larger than 2 MDa, comprises more than fifty different polypeptides, including RNA polymerase II, GTFs, Mediator [4-6]. GTF2D consists of the TATA Box – Binding Protein (TBP) and TBP – Associated Factors (TAFs), a group of evolutionarily conserved proteins that participate in determining the state of chromatin, contribute to promoter recognition, serve as coactivators, and post-translationally modify other GTA proteins to facilitate PIC assembly and transcription initiation [1,5]. Its DNA binding activity is regulated by positive and negative cofactors such as heterodimeric NC2 (also named Dr1/Drap 1), comprised of α- and β-type subunits [7,8]. The molecular actions of GTA proteins were clarified through an extensive series of studies [1-3,6,9]. On the other hand, only scanty success was obtained in identifying their biological functions as well as in verifying their involvement in genetic pathology, including tumorigenesis [10,11]. We examined the involvement of GTA proteins in the pathogenesis of Neuroblastoma (NB), exploiting our data and those from the literature on the genomics of GTA (see Additional file 1; reviewed in ref. [12]) to perform the positional and functional gene candidate approach. NB is a group of early childhood tumours with a complex molecular pathogenesis [13,14]. It is generally believed that the abnormally proliferating cell in all types of NB is the neuroblast, a fleeting stem cell that transiently appears during the early stages of mammalian development [15]. NB clinical phenotype is remarkably heterogeneous, ranging from spontaneous regression to restless progression [14,16]. This variability is associated to a high genetic heterogeneity. The most important genomic alterations in NB are interstitial deletions at 1p, 11q, 17q, and MYCN amplification [14,17]. NB molecular phenotype is characterized by the altered expression of a plethora of genes belonging to different Gene Ontology categories: all of them are potential NB biomarkers [14,18]. Our analysis was initially focused on human chromosome 1 short arm, where one or more NB master genes are thought to reside [17,19]. In this region, we had previously mapped the genes encoding TAF13, GTF2B, NC2β, TAF12 to 1p13.3, 1p21-p22, 1p22.1, 1p35.3, respectively [20-22].

## Methods

### NB samples

NB patients were 30 males and 28 females. Tumour primary site was adrenal in 33 patients, abdominal nonadrenal in 18, thoracic in 5, cervical in 2. Tumours were classified according to the International Neuroblastoma Pathology Classification (INPC) [23]. The final pathologic diagnosis fulfilled the International Criteria for Neuroblastoma Diagnosis [13]. Patients were staged according to the International Neuroblastoma Staging System: 12 patients were at stage I, 8 at stage II, 6 at stage III, 23 at stage IV, and 9 at stage IVS. The clinical and molecular characteristics of the patients are reported (see Additional file 2).

### Expression of TAF13, GTF2B, NC2α, TAF12, NC2β

Total RNA from NB biopsies and from peripheral blood of ten normal controls (BL) was isolated according to our published protocol [12]. Primers were designed with the OMIGA 2.0 software (Oxford Molecular) by using as template the published sequences of *Homo sapiens *genes: their sequence and corresponding RT-PCR conditions are shown in Table 1. Transcript reduction was determined by calculating the arithmetic mean of the densitometric values of all samples and using this value as cut-off: only differences that were at least threefold were considered significant to this study.

**Table 1:** Primers and RT-PCR conditions for expression analysis

**PCR primers**	**RT-PCR conditions**
NC2β fw: 5' CGATGATGATCTCACTATCC 3' NC2β rev: 5' GTTGCTGTCTAGCTTTTGC 3'	50°C 30 min; (94°C 60 sec, 47°C 90 sec, 72°C 2 min) 25×; 72°C 10 min.
NC2α fw: 5' AGACGGACGAAGAGATTGG 3' NC2α rev: 5' CATGTCGGGAACAGATGC 3'	50°C 30 min; (94°C 60 sec, 53°C 90 sec, 72°C 2 min) 27×; 72°C 10 min.
GTF2B fw: 5' AGAAGAGCCTGAAGGGAAGAGC 3' GTF2B rev: 5' CAGCAACACCAGCAATATCTCC 3'	50°C 30 min; (94°C 60 sec, 50°C 90 sec, 72°C 2 min) 27×; 72°C 10 min.
TAF12 fw: 5' GAGCAGTTGGATGAAGATGTGG 3' TAF12 rev: 5' TGAGATGGCAGGGAAAAGG 3'	50°C 30 min; (94°C 60 sec, 57°C 90 sec, 72°C 2 min) 26×; 72°C 10 min.
TAF13 fw: 5' GCAGATGAGGAAGAAGACC 3' TAF13 rev: 5' TATCTTCAACTTGTACTCGACC 3'	50°C 30 min; (94°C 60 sec, 54°C 90 sec, 72°C 2 min) 27×; 72°C 10 min.
β actin fw: 5' GTGCCCATCTATGAGGGTTACG 3' β actin rev: 5' TGATCCACATCTGCTGGAAGG 3'	50°C 30 min; (94°C 60 sec, 46°C 90 sec, 72°C 2 min) 25×; 72°C 10 min.

### Genomics of TAF13, NC2β, TAF12 in NB samples

**Figure 1:**
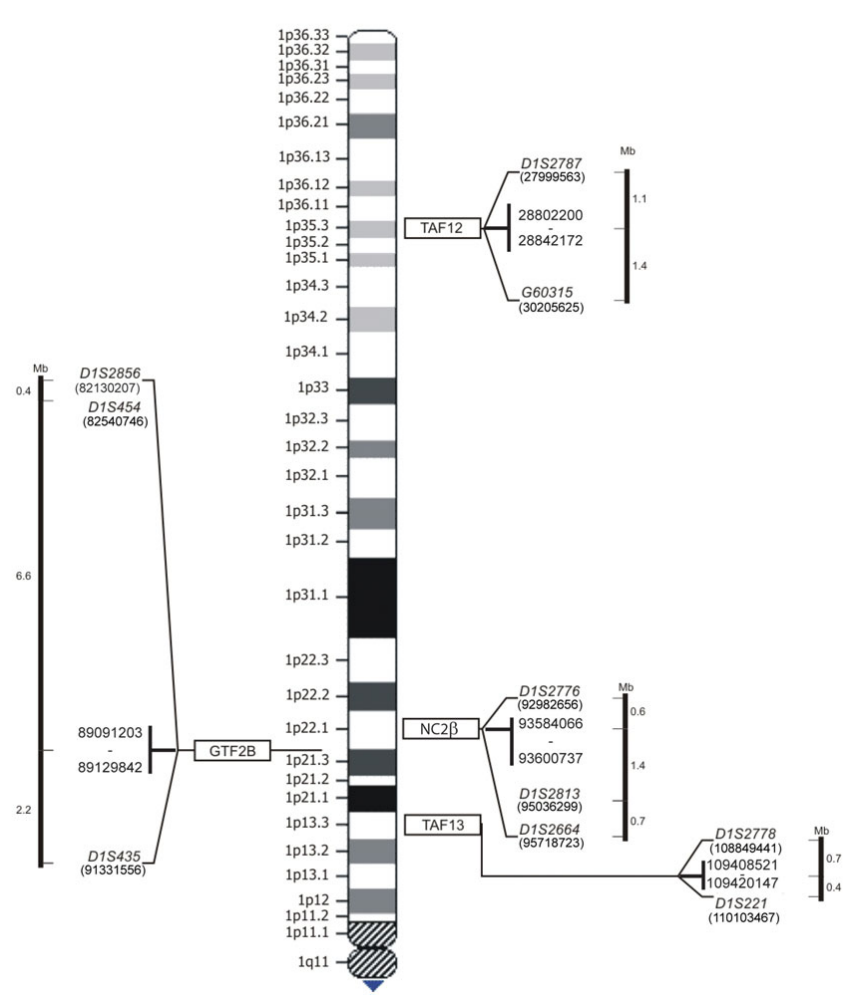
**Genomics of TAF13, GTF2B, NC2β, TAF12**. Genomic position of TAF13, GTF2B, NC2β, TAF12 and corresponding microsatellite polymorphic markers.

Microsatellite polymorphic markers were selected from the Marshfield map [24] as close as possible to the 5' and 3' ends of TAF13, GTF2B, NC2β, TAF12 (Table 2, Figure 1). Primers for sequencing were designed with the same software; their sequence was: NC2βS: gtgggtgggggaagg; TAF12S: agggtgtatttatatatagttta; TAF13S: tcccaactaattacactact. PCR was performed according to standard protocols (Invitrogen). Tumour and constitutional (blood) DNA (3 to 5 ng depending on primers efficiency) was amplified with a Master Cycler Gradient (Eppendorf). Forward primers were labeled at their 5' with the fluorochromes Tamra, Joe or 6-Fam (MWG Biotech). Amplified DNA was loaded on ABI PRISM™ 310 Genetic Analyzer (Applied Biosystems): the results were analyzed with ABI PRISM 310 GeneScan 3.1 software. GI was determined by comparing the allelic ratios between constitutional and tumour DNA in heterozygous samples [25]. Theoretically QGI = 0 expresses the total loss of one allele and QGI = 1 indicates the normal allele ratio. We refer to *Genomic Imbalance *when QGI ≤ 0.5.

**Table 2:** Microsatellite polymorphic markers used for GI analysis

**Locus**	**Marker**	**Chromosomal Position**	**Heterozygosity**	**PCR primers**	**Size (bp)**	**PCR conditions**
**NC2β 5'**	D1S2776	92982656 – 92982869	0.75	5' AATGCCTGTCTTTATCCCTG 3' 5' AATGTAAGAGAAATGCCCCT 3'	196 – 212	95°C 10 min (95°C 30 sec, 52°C 75 sec, 72°C 30 sec) 30×; 72°C 10 min
**NC2β 3'**	D1S2813	95036107 – 95036299	0.72	5' CTTTTGACTCACTGGAAGACAT 3' 5' CCCCACCGTATCTGGTAT 3'	185 – 205	95°C 10 min (95°C 30 sec, 55°C 75 sec, 72°C 30 sec) 30×; 72°C 10 min
**NC2β 3'**	D1S2664	95718483 – 95718723	0.73	5' CAGCCCACAGAATAACACTG 3' 5' TTCATGCTATGATTTTCCGC 3'	202 – 254	95°C 10 min (95°C 30 sec, 54°C 75 sec, 72°C 30 sec) 30×; 72°C 10 min
**TAF12 5'**	D1S2787	27999563 – 27999728	0.76	5' TTTAACCCTGGAAGGTTGAG 3' 5' ACAGGACAATGCTGTCAGTATG 3'	137 – 167	95°C 10 min (95°C 30 sec, 52°C 75 sec, 72°C 30 sec) 30×; 72°C 10 min
**TAF12 3'**	G60315	30205319 – 30205625	-	5' AGCTGAGTCAGGGAAACCCATT 3' 5' TGTGCTCTTCAATGTGTTAGGGA 3'	307 – 340	95°C 10 min (95°C 30 sec, 56°C 75 sec, 72°C 30 sec) 30×; 72°C 10 min
**TAF13 5'**	D1S2778	108849441 – 108849609	0.66	5' CACAGTTAAATTGCATTTCC 3' 5' GCTCACCATAAACAAGAGG 3'	161 – 173	95°C 10 min (95°C 30 sec, 50°C 75 sec, 72°C 30 sec) 30×; 72°C 10 min
**TAF13 3'**	D1S221	110103235 – 110103467	0.75	5' CCTACAACTCCATCCTGTCC 3' 5' GTCTTAAGTCGCTCTGCCTG 3'	215 – 225	95°C 10 min (95°C 30 sec, 56°C 75 sec, 72°C 30 sec) 30×; 72°C 10 min
**NC2α 3'**	D11S1889	67069719 – 67069901	0.67	5' AGCTGGACTCTCACAGAATG 3' 5'CAAGAGGCTGGTAGAAGGTG3'	183 – 207	95°C 10 min (95°C 30 sec, 60°C 30 sec, 72°C 30 sec) 30×; 72°C 10 min
**NC2α 3'**	D11S4178	67945684 – 67945935	0.69	5' CAGGCCCAGTCTCTTG 3' 5' CGTGTCCAGATGAAAGTG 3'	237 – 260	95°C 10 min (95°C 30 sec, 52°C 75 sec, 72°C 30 sec) 30×; 72°C 10 min.
**GTF2B 5'**	D1S2856	82130207 – 82130465	0.5	5' AGCTCTGTGACATTGGATAA 3' 5' CAGAACATAATAAGTGTGGCTA 3'	257 – 263	95°C 10 min (95°C 30 sec, 53°C 75 sec, 72°C 30 sec) 30×; 72°C 10 min.
**GTF2B 5'**	D1S454	82540746 – 82540872	0.56	5' TGTTAGTTCCTGTTCTTGGTGA 3' 5' TTCCCTGGAAACAACCATAA 3'	155 – 163	95°C 10 min (95°C 30 sec, 58°C 75 sec, 72°C 30 sec) 30×; 72°C 10 min
**GTF2B 3'**	D1S435	91331437 – 91331556	0.73	5' GGCCACATGGGAATTTTCT 3' 5' AGCAGTTCAAGGCCACAGT 3'	157 – 177	95°C 10 min (95°C 30 sec, 55°C 75 sec, 72°C 30 sec) 30×; 72°C 10 min

### Methylation analysis

The location of CpG islands in the *bone fide *promoter regions of NC2β, TAF12 and TAF13 was determined with the CpGPLOT software [26], after masking the repeated sequences with Repeat Masker [27]. The NB samples and cell lines analyzed are listed in Additional file 3. Quantitative methylation analysis was performed by pyrosequencing with a SPQ 96MA instrument (Biotage, Uppsala, Sweden) [28]. Pyrosequencing is a sequencing by synthesis-analysis of short genomic sequences that is ideally suited for SNP analysis. In this respect, DNA methylation can be considered a special case of polymorphism revealed by the bisulfite chemical reaction that converts only the unmethylated Cs into Ts [29]. 2 μl of bisulfite modified DNA were amplified with primers designed with the Assay Design Software for Pyrosequencing (Biotage, Uppsala, Sweden) to amplify target sequences independently of their methylation status. The amplified targets were then subjected to pyrosequencing analysis. Primers sequences were: NC2βF: gtttttgtgaaggaatggga; NC2βR: tcaaatttccccctccct (amplicon size: 153 bp, Ta 58°C); TAF12F: aagagtaagttgtagggtgtattt; TAF12R: acaaaaactaccccaataaaa (amplicon size: 214 bp, Ta 58°C); TAF13F: ggtttttttttttagagattgt; TAF13R: aaaatcttcttcctcatctactacca (amplicon size: 208 bp, Ta 58.5°C). Sequencing reactions were performed with the Pyro Gold reagent kit SPQ 96MA according to the manufacturer instructions (Biotage, Uppsala, Sweden).

### Statistical analysis

We correlated our data by using an A-Priori data mining algorithm for tables with Unknown Values corresponding to missing data [30]. Let T (A1, A2, ..., Ak) be a table with attributes (columns) A1, A2, ..., Ak. Each column Ai is a predicate (truth-value function) that assigns to every row r (experiment) one of the three values: True, False, Unknown. This means that Ai(r) = True/False/Unknown indicates that Ai holds/does not hold/is unknown in the experiment r. For example, rows may be NB samples and columns may be RT-PCR or GI data. In this case, Ai(r) = True (resp. False, resp. Unknown) may indicate that gene Ai is overexpressed (resp. is not overexpressed, resp. could not be unambiguously assigned a value) in sample r. Datamining searches for highly related facts that constitute a frequent itemset. Any datamining algorithm tries to construct the collection Lj of all frequent itemsets S of size j.

#### Definition 1

(frequent itemset). Given a positive threshold t, a frequent itemset S of size j includes j columns Ai1, Ai2, ..., Aij such that the ratio [Number of rows r in which (Ai1(r) AND*Ai2(r) AND* ... AND* Aij(r)) = True]/[Number of rows r in which (Ai1(r) AND* Ai2(r) AND* ... AND* Aij(r)) ≠ UNKNOWN] > t.

The commutative three-valued logical conjunction AND* is an extension of the classical logical conjunction by the following table rows:

A B A AND* B

True Unknown Unknown

False Unknown False

Unknown Unknown Unknown

The A-priori datamining algorithm exploits the monotonicity property: this in the classic two-valued logic (true/false) implies that subsets of frequent sets are themselves frequent. However, in the presence of Unknown Values this notion must be slightly modified in the following way.

#### Definition 2

(candidate set). A set S of j columns Ai1, Ai2, ..., Aij is candidate if

[Number of rows r in which (Ai1(r) AND* Ai2(r) AND* ... AND* Aij(r)) = True]/[Number of rows r in which there are NOT UNKNOWN values] >t.

Here is the pseudo-code for the A-priori data mining with unknown values

Inizialization step:

Let C1 be the set of candidate single columns.

Let L1 be the set of columns Ai in C1 which are also frequent.

Recursive step:

For each j >1

Let Cj be the set of j columns Ai1, Ai2, ..., Aij, such that each subset of j-1 columns is in Cj-1.

Let Lj be the collection of sets in Cj that are frequent.

Since in practice very few rows have Unknown Values, the speed-up given by the above A-priori strategy is similar to that given by the classical two-valued logic A-priori datamining. To establish the statistical significance of our results, p-values were computed by using Efron multiple hypotheses testing [31].

## Results and Discussion

**Figure 2:**
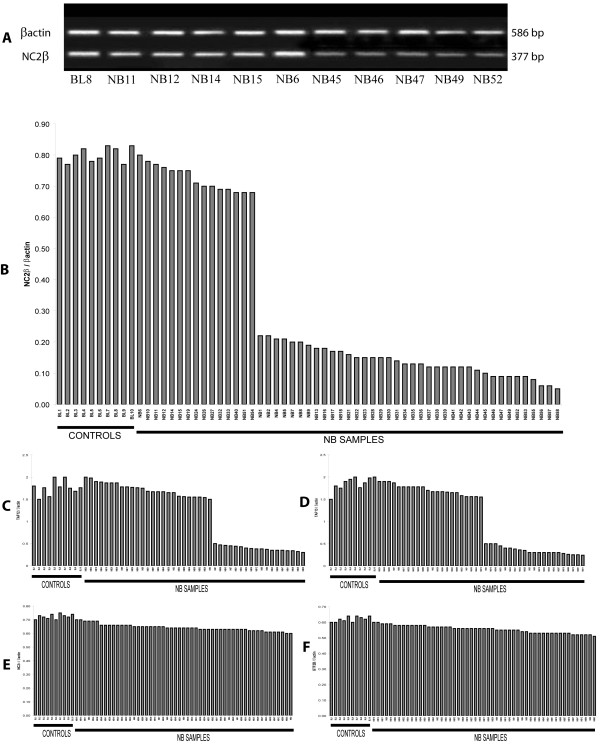
**TAF13, GTF2B, NC2α, TAF12, NC2βexpression in NB samples**. A: RT-PCR analysis of NC2β expression in a control (BL8) and in NB samples. B, C, D: Diminished expression of NC2β, TAF12, TAF13 in NB samples with respect to human adult peripheral blood (BL). E, F: Similar expression of GTF2B and NC2α in control and NB samples.

**Figure 3:**
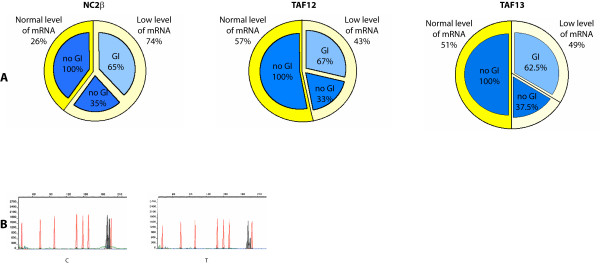
**Genotype/phenotype correlation in neuroblastoma**. A: Normal levels of the mRNAs encoding NC2β, TAF12, TAF13 always correspond to GI absence. Low levels of these mRNA species are frequently associated with GI. B: Densitometric pattern of microsatellites showing GI at the locus NC2β in a NB sample (T) and its absence in the blood (C) from the same individual.

**Figure 4:**
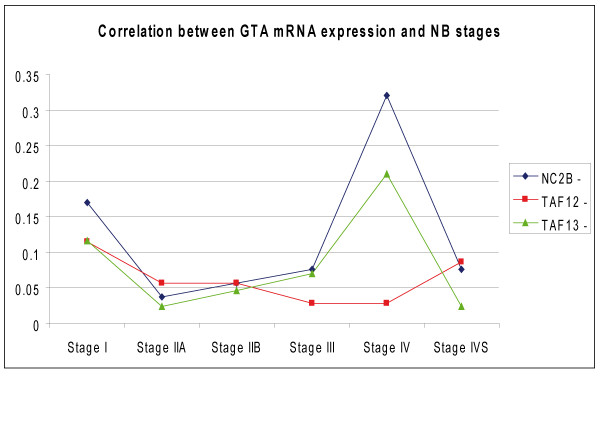
**Correlation between GTA mRNA expression and NB stages**. A statistically significant association exists between NC2β mRNA decrease and NB stages III and IV (4/39 and 17/39, respectively) (p = 0.003937). Interestingly, it was also found with stage I (p = 0.003922): this could suggest a stage-specific selection of the NC2β (-) mRNA phenotype.

**Figure 5:**
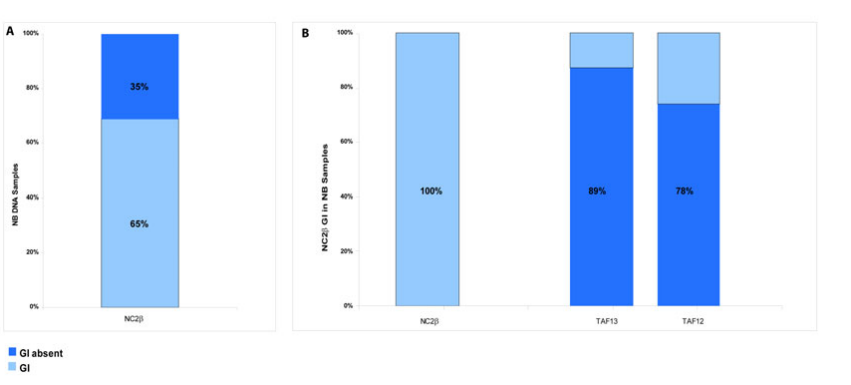
**GI frequency at the loci TAF13, NC2β, TAF12**. In NB samples with Genomic Imbalance (GI) for NC2β, we never detect it for TAF12 or TAF13: the anticorrelation between NC2β and TAF13 reached statistical significance (p = 0.043). TAF 12 and TAF 13 seemed to show a reciprocal positive correlation since in 80% of samples GI was absent in both.

**Figure 6:**
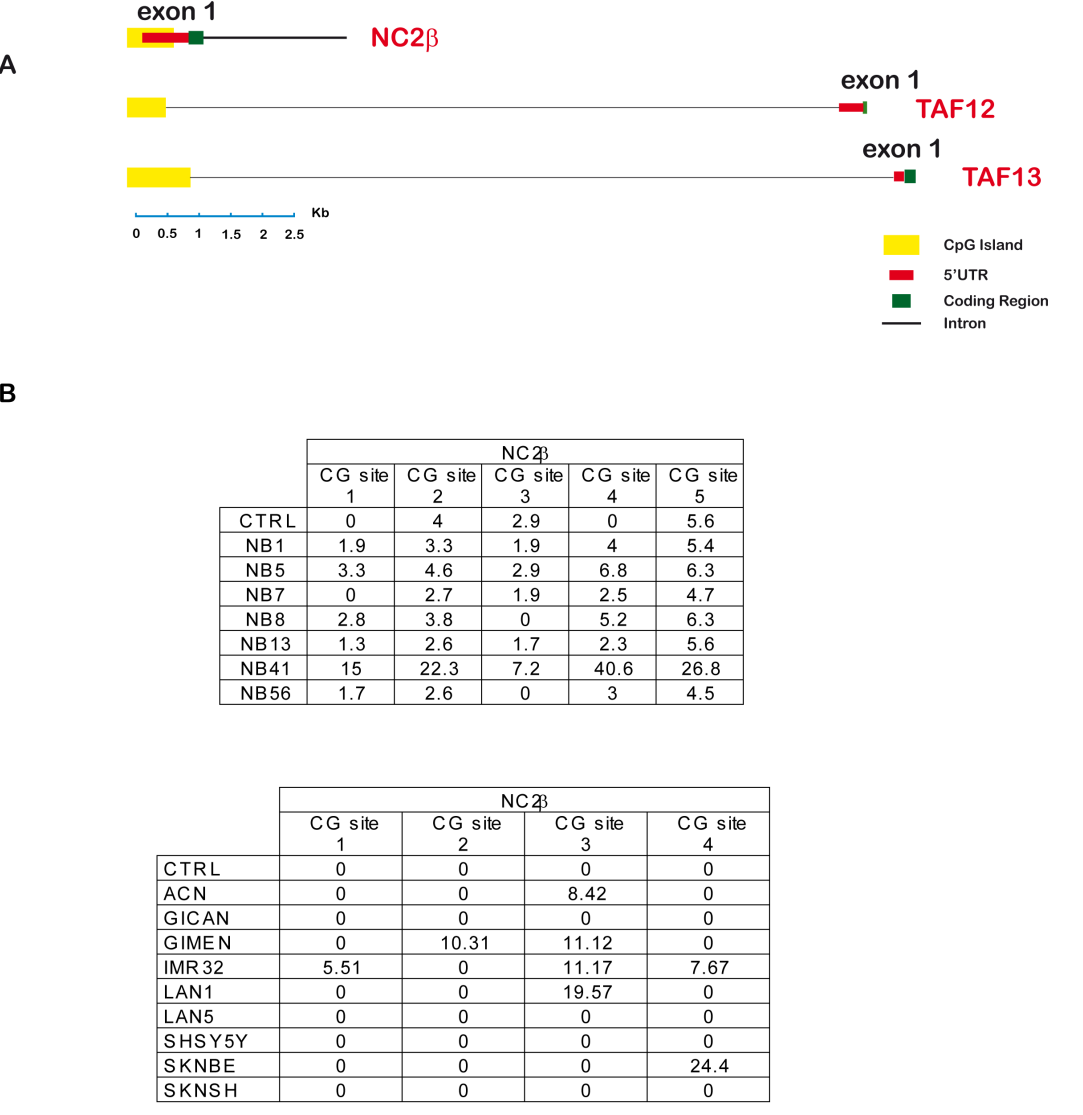
**Methylation of NC2β promoter**. A: CpG islands upstream and downstream of NC2β, TAF12, TAF13 transcription start site. B: Methylation percentage of CpG sites within NC2β island. Samples with methylation levels below 10% are considered unmethylated: accordingly, we suggest that promoter methylation may explain the low NC2β mRNA expression only in sample NB41 (Figure 2). For methylation analysis of TAF12 and TAF13 promoter, (see Additional file 3).

Based on TAF13, GTF2B, NC2β, TAF12 genomic location and molecular functions, we considered these four GTA genes as positional and functional candidates for involvement in NB pathogenesis (see Additional file 1, Table 1 and Figure 1). As reference marker, we used the GTA protein NC2α (the molecular partner of NC2β), that is encoded by a gene located at 11q13.3 (see Additional file 1, Table 1) [32]. Expression of these genes was analyzed through RT – PCR in fifty eight NB biopsies (see Additional file 2, Table 1 and Figure 2). As a preliminary step, we characterized their mRNA phenotype in ten peripheral blood samples from normal individuals and we did not observe significant variations of their transcription level (Table 1, Figure 2). On the other hand, in NB we found a clear-cut decrease (from 3 to 8 times) of the mRNAs encoding TAF13, NC2β, TAF12 with respect to peripheral blood from the same individuals in 49% (21/43), 74% (39/53), 43% (19/44) samples, respectively (Figures 2 and 3). Statistical analysis demonstrated a significant positive correlation between NC2β transcripts reduction and NB (p = 0.0039), whereas this was not obtained for TAF13 and TAF12 (Table 3). NC2β mRNA levels are significantly decreased in both NB advanced stages III and IV (p = 0.0039); their reduction also in stage I (p = 0.0039) could suggest a peculiar natural history of NB (Figure 4). In the same set of samples, we did not detect any change of the levels of the mRNAs encoding GTF2B and NC2α (Figure 2). By using at least two closely flanking microsatellite markers for each gene, we then analyzed the genome of the same NB samples for deletions at the loci TAF13, GTF2B, NC2β, TAF12, NC2α (Table 2, Figures 1 and 3): these were found in more than 60% of samples with diminished levels of the mRNAs encoding either TAF13 (62.5%), NC2β(65%), or TAF12 (67%), but were never detected in NB samples with normal levels of mRNA for these three genes (Figures 3A, B, 5A). Similarly, we never found deletions at the GTF2B and NC2α loci both in NB biopsies as well as in controls (not shown). Due to aneuploidy, we suggest that for NB as for other tumours GI (Genomic Imbalance) may be more appropriate than LOH (Loss of Heterozygosity). In NB samples with GI for NC2β, we never detect it for TAF12 or TAF13 (Figure 5B): the anticorrelation between NC2β and TAF13 reached statistical significance (p = 0.043). TAF 12 and TAF 13 seemed to show a reciprocal positive correlation since in 80% of samples GI was absent in both. Besides genomic deletions, the reduced levels of the mRNAs encoding NC2β, TAF12, TAF13 in NB samples could be due to: (i) epigenetic modifications of promoter DNA, such as methylation [33]; (ii) mutations in the promoter or other regulatory regions [34]; (iii) increased mRNA turnover [35]; (iv) negative regulation by miRNAs [36,37]. To test the first of these possibilities, we analyzed a panel of NB biopsies and NB cell lines with low levels of mRNAs for these three genes and checked the methylation status of the 4 to 7 CpG doublets found in their *bona fide *promoter (see Additional file 3, Figure 6). In general, very low or no methylation was observed both in the tumor samples as in the cell lines (see Additional file 3, Figure 6). We did observe higher levels of DNA methylation in the NC2β gene, that was however not correlated to its expression with the exception of sample NB41 (Figures 2 and 6): this indicates that other mechanisms, such as negative regulation by microRNAs, should be considered to account for the low levels of expression of these genes in NB [36,37]. Our data allow us to speculate on the biological role of GTA proteins NC2, TAF12, TAF13, GTF2B. NC2 is an evolutionarily conserved transcriptional regulator for which an intriguing general role was proposed: it could repress basal transcription in the absence of activators or alternatively stimulate it in their presence [9]. Analogously to p53 role as *Guardian of the Genome*, NC2 could represent the *Guardian of the Transcription Machinery *and control its balanced functioning, performing a role as tumor suppressor possibly not restricted to NB: reduced levels of the protein could be responsible for the inappropriate activation of genes promoting cellular proliferation, thus contributing to cell transformation. Its two subunits NC2α and NC2β could perform distinct roles in the regulation of gene expression and determination of cell phenotype: binding of holoNC2 marks repressed promoters, while occupancy by NC2α correlates with active promoters [38]. TAF12 and TAF13 encode two small subunits of GTF2D. TAF12 interacts directly with TBP [39]. Similar to TAF4b, TAF6 and TAF9, it contains a histone fold domain (HFD): the polypeptide is part of a histone-like TAF complex that was shown to be critically important for GTF2D architecture [40]. TAF13, that interacts with TBP, TAF10 and TAF11, is associated with only a subset of GTF2D complexes [39]. Reduced levels of TAF12 and TAF13 could alter GTF2D structure and function and deregulate the expression of different genes, such as those involved in cell cycle control: different mutations of yTAF25 (the yeast ortholog of TAF12) are known to induce distinct phenotypes and affect the regulation of different subsets of genes [41]. GTF2B is a highly conserved member of GTA [42]: it forms a complex with GTF2D and GTF2A (the DAB complex), bridging RNA polymerase II and the promoter [43]. The protein is also a target of Mediator and gene – specific transcriptional activators [44]. Its critical molecular and biological role could explain our failure to detect GTF2B deletions in NB, since they would expose the cells to a strong negative selection. A similar argument could be put forward to explain the absence of NC2α deletions in our dataset. Cell proliferation and specularly tumorigenesis are very complex phenomena. Aneuploidy and GI are common features of neoplastic genomes [45,46]. The ensuing haploinsufficiency may cause loss of the cell ability to autonomously control its proliferation and to coordinate it with that of other cells of the same organism. Unexpected odd partners seem to be involved in the process. Our approach has allowed us to demonstrate that reduced expression and GI at the GTA locus NC2β is frequently and specifically present in the genome of NB tumour cells, possibly as a result of mutations of *caretaker *genes involved in DNA repair or chromosomal segregation and of complex selective mechanisms [14, 46, this paper]. The possible anticorrelation between NC2β on the one side and TAF12 and TAF13 on the other may suggest that these proteins could perform analogous biological roles and as such they may reciprocally complement each other as negative regulators of proliferation.

**Table 3:** Correlation between decrease of GTA genes mRNA and NB phenotype

	**NC2β**	**TAF13**	**TAF12**
**NB patients with mRNA decrease**	73.6% (39/53)	48.8% (21/43)	43.2% (19/44)
**NB patients with normal mRNA levels**	26.4% (14/53)	51.2% (22/43)	56.8% (25/44)
**p-value**	**0.0039**	0.543	0.527

## Conclusion

The data presented in this paper experimentally confirm our hypothesis that at least some GTA proteins may also be physiologically involved in the control of cell proliferation, at the same time underscoring the importance of natural selection within complex biopathological processes [22,47]. They also suggest possible ways to exploit molecular *omic *profiling to determine biological functions and design rational anticancer therapies.

## Competing interests

The authors declare that they have no competing interests.

## Authors' contributions

MP conceived and directed the project. CdP, MR (Ge), GPT, AdC, KHG, AF, AG designed some of the experiments. MR (CT), DB, LRD, MRG, AM, VG, AR, ET, MM, BB, ID, SF, AL, CB, RG, AP carried out experiments. MP and MR (Ge) wrote the paper.

## Supplementary Material

Additional file 1 Genomics and Transcriptomics of Human GTA. Click here for file

Additional file 2 NB Patients: Clinical, Pathological, Biomolecular Data. Click here for file

Additional file 3 Methylation of TAF12 and TAF13 in NB biopsies and NB cell lines. Click here for file
