# Involvement of GTA protein NC2beta in Neuroblastoma pathogenesis suggests that it physiologically participates in the regulation of cell proliferation

**DOI:** 10.1186/1476-4598-7-59

**Published:** 2008-06-30

**Authors:** Cinzia Di Pietro, Marco Ragusa, Davide Barbagallo, Laura R Duro, Maria R Guglielmino, Alessandra Majorana, Veronica Giunta, Antonella Rapisarda, Elisa Tricarichi, Marco Miceli, Rosario Angelica, Agata Grillo, Barbara Banelli, Isabella Defferari, Stefano Forte, Alessandro Laganà, Camillo Bosco, Rosalba Giugno, Alfredo Pulvirenti, Alfredo Ferro, Karl H Grzeschik, Andrea Di Cataldo, Gian P Tonini, Massimo Romani, Michele Purrello

**Affiliations:** 1 Dipartimento di Scienze Biomediche, Sezione di Biologia Generale, Biologia Cellulare, Genetica Molecolare *G Sichel*, Unità di Biologia Genomica e dei Sistemi Complessi, Genetica, Bioinformatica, Università di Catania, 95123 Catania, Italy; 2 Labogen, 95124 Catania, Italy; 3 Istituto Nazionale per la Ricerca sul Cancro (IST), Sezione di Genetica dei Tumori, 16132 Genova, Italy; 4 Istituto Nazionale per la Ricerca sul Cancro (IST), Sezione di Oncologia Traslazionale Pediatrica, 16132 Genova, Italy; 5 Dipartimento di Matematica ed Informatica, Università di Catania, 95123 Catania, Italy; 6 Medizinisches Zentrum für Humangenetik, Philipps Universität, 35037 Marburg, Germany; 7 Dipartimento di Pediatria, Università di Catania, 95123 Catania, Italy

## 

After publication of this work [1], we noticed that as Dr Rosario Angelica was added as an author late in the process, details were inadvertently omitted from the "Authors' contributions" and "Competing interests" sections of the article. These have now been modified accordingly.

## Competing interests

The authors declare that they have no competing interests.

## Authors' contributions

MP conceived and directed the project. CdP, MR (Ge), GPT, AdC, KHG, AF, AG designed some of the experiments. MR (CT), DB, LRD, MRG, AM, RA, VG, AR, ET, MM, BB, ID, SF, AL, CB, RG, AP carried out experiments. MP and MR (Ge) wrote the paper.
