# Supplementary material for: Involvement of GTA protein NC2β in Neuroblastoma pathogenesis suggests that it physiologically participates in the regulation of cell proliferation
Source: Mol Cancer. 2008 Jun 6;7:52. doi: 10.1186/1476-4598-7-52 (PMC2443168; doi:10.1186/1476-4598-7-52)
Supplement: Additional file 3 — Methylation of TAF12 and TAF13 in NB biopsies and NB cell lines. [file 1476-4598-7-52-S3.doc]

**Table 3s**

**Methylation of TAF12 and TAF13 in NB biopsies and NB cell lines**

|  | **TAF12** | | | | | |
| --- | --- | --- | --- | --- | --- | --- |
|  | CG site 1 | CG site 2 | CG site 3 | CG site 4 | CG site 5 | CG site 6 |
| CTRL | 0 | 0 | 0 | 0 | 0 | 0 |
| NB1 | 0 | 3.1 | 0 | 0 | 0 | 0 |
| NB5 | 3.9 | 0 | 0 | 0 | 0 | 0 |
| NB7 | 2.8 | 3.4 | 0 | 0 | 0 | 0 |
| NB8 | 4 | 5 | 6.2 | 0 | 0 | 0 |
| NB13 | 0 | 0 | 0 | 0 | 0 | 0 |
| NB41 | 0 | 4.5 | 0 | 0 | 0 | 0 |
| NB56 | 0 | 0 | 0 | 0 | 0 | 0 |

|  | **TAF12** | | | | |
| --- | --- | --- | --- | --- | --- |
|  | CG site1 | CG site2 | CG site3 | CG site4 | CG site5 |
| CTRL | 0 | 0 | 0 | 0 | 0 |
| ACN | 0 | 0 | 12.8 | 0 | 0 |
| GICAN | 0 | 0 | 0 | 0 | 0 |
| GIMEN | 0 | 0 | 0 | 0 | 0 |
| IMR32 | 0 | 0 | 0 | 0 | 0 |
| LAN1 | 0 | 0 | 0 | 0 | 0 |
| LAN5 | 9.3 | 0 | 0 | 0 | 0 |
| SHSY5Y | 0 | 0 | 0 | 0 | 0 |
| SKNBE | 0 | 0 | 0 | 0 | 0 |
| SKNSH | 10.1 | 0 | 0 | 0 | 0 |

|  | **TAF13** | | | | | | |
| --- | --- | --- | --- | --- | --- | --- | --- |
|  | CG site 1 | CG site 2 | CG site 3 | CG site 4 | CG site 5 | CG site 6 | CG site 7 |
| CTRL | 0 | 0 | 0 | 0 | 0 | 0 | 8.3 |
| NB1 | 0 | 0 | 3 | 0 | 0 | 0 | 0 |
| NB5 | 3.3 | 0 | 0 | 0 | 0 | 0 | 0 |
| NB7 | 0 | 3 | 0 | 0 | 0 | 0 | 0 |
| NB8 | 0 | 3.9 | 0 | 0 | 0 | 0 | 0 |
| NB13 | 2.3 | 2.1 | 2.2 | 1.4 | 0 | 0 | 4.1 |
| NB41 | 0 | 0 | 0 | 0 | 0 | 0 | 0 |
| NB56 | 0 | 0 | 0 | 0 | 0 | 0 | 0 |

|  | **TAF13** | | | | | |
| --- | --- | --- | --- | --- | --- | --- |
|  | CG site1 | CG site2 | CG site3 | CG site4 | CG site5 | CG site6 |
| CTRL | 0 | 0 | 0 | 0 | 0 | 0 |
| ACN | 0 | 0 | 0 | 0 | 0 | 0 |
| GICAN | 0 | 0 | 0 | 0 | 0 | 0 |
| GIMEN | 0 | 0 | 0 | 0 | 0 | 0 |
| IMR32 | 0 | 0 | 0 | 0 | 0 | 0 |
| LAN1 | 0 | 0 | 0 | 0 | 0 | 0 |
| LAN5 | 0 | 0 | 0 | 0 | 0 | 0 |
| SHSY5Y | 0 | 0 | 0 | 0 | 0 | 0 |
| SKNBE | 0 | 0 | 0 | 0 | 0 | 0 |
| SKNSH | 0 | 0 | 0 | 0 | 0 | 0 |
